# Effects of heat stress on body temperature, milk production, and reproduction in dairy cows: a novel idea for monitoring and evaluation of heat stress — A review

**DOI:** 10.5713/ajas.18.0743

**Published:** 2019-01-04

**Authors:** Jiangjing Liu, Lanqi Li, Xiaoli Chen, Yongqiang Lu, Dong Wang

**Affiliations:** 1 Institute of Animal Science, Chinese Academy of Agricultural Sciences, Beijing 100193, China; 2 Department of Animal Genetics, Breeding and Reproduction, College of Animal Science and Technology, Jilin Agricultural University, Changchun 130118, China; 3 Animal Husbandry Station of Beijing, Beijing 100107, China

**Keywords:** Body Temperature, Dairy Cows, Heat Stress, Temperature-humidity Index

## Abstract

Heat stress exerts a substantial effect on dairy production. The temperature and humidity index (THI) is widely used to assess heat stress in dairy operations. Herein, we review the effects of high temperature and humidity on body temperature, feed intake, milk production, follicle development, estrous behavior, and pregnancy in dairy cows. Analyses of the effects of THI on dairy production have shown that body temperature is an important physiological parameter in the evaluation of the health state of dairy cows. Although THI is an important environmental index and can help to infer the degree of heat stress, it does not reflect the physiological changes experienced by dairy cows undergoing heat stress. However, the simultaneous measurement of THI and physiological indexes (e.g., body temperature) would be very useful for improving dairy production. The successful development of automatic detection techniques makes it possible to combine THI with other physiological indexes (i.e., body temperature and activity), which could help us to comprehensively evaluate heat stress in dairy cows and provide important technical support to effectively prevent heat stress.

## INTRODUCTION

Heat stress is a non-specific physiological response of animal to thermal environment when animal produces more heat than it can dissipate [1]. The Holstein is the most popular dairy cattle breed in the world. In summer, the ability of this breed to dissipate heat via skin evaporation is limited by its relatively low surface area to body weight ratio, underdeveloped sweat glands, and short, dense body surface hair, all of which burden milk production [2]. Moreover, considerable metabolic heat is produced by the fermentation of the consumed roughage in the rumen, which also results in an additional heat load on the cow’s body [3]. In addition, with the steadily warming global climate and the intensification of hot periods, dairy cows have been subjected to even more severe heat stress [4,5], which threatens to severely reduce production performance, especially milk yield [6,7]. Therefore, dairy cows used to mainly be raised in cool areas to reduce heat stress. To help mitigate the negative effects of ambient temperature and humidity on body temperature and milk production, studies have focused on the development of scientific techniques to manage heat stress and increase cooling [4,8]. These techniques can reduce the negative impact of hot and humid environments on dairy cows, and promote increases in the quality and efficiency of milk production in the dairy industry [9].

Body temperature is an important physiological parameter and is indicative of the health of dairy cows [10]. Studies have shown that high temperature conditions can lead to significant increases in body temperature and respiratory rate [11,12], and a significant decrease in feed intake [9], milk production [13,14], and reproductive performance [15]. The annual economic losses of livestock industries as a result of heat stress have been reported to be 1.69 to 2.36 billion USD in the United States, of these losses, up to 1,500 million USD occur in the dairy industry [16]. These data show that the most serious economic impact of heat stress has been on the dairy industry. Thus, if real-time changes in record body temperature, ambient temperature, and humidity can be monitored and recorded, it would be possible to evaluate whether cows are experiencing heat stress, as well as the degree of heat stress they are experiencing, based on these changes [17,18].

The temperature-humidity index (THI) is often used as an important environmental assessment index to evaluate heat stress in dairy production [19,20]. Studies that focus on the relationship between THI and body temperature will help us to understand the physiological status of cows exposed to different THI conditions, which is of great significance to the feeding and management of cows. Herein, we review the effects of ambient temperature and humidity on the body temperature of cows, as well as the effects of heat stress on feed intake, milk production, follicular development, estrus, and pregnancy. We hope that this review will provide theoretical guidance for the feeding and management of dairy cows.

## EFFECTS OF HEAT STRESS ON THE BODY TEMPERATURE OF DAIRY COWS

### Formulas for calculating temperature-humidity index

One study indicated that animals show different sensitivities to ambient temperature and humidity [15]. Under the same ambient humidity, swine cannot tolerate higher temperatures because they have few sweat glands [21], while the impact of heat stress on cattle can be relieved by dissipating excessive heat through sweating [20]. Thus, THI can be used as an important environmental index to ascertain whether animals are in a state of heat stress, as well as the degree of this heat stress. THI—a comprehensive index composed of the effects of ambient temperature and humidity—has been developed into a meteorological safety index to monitor the possible effects of ambient temperature and humidity on animals [22]. It has been shown the degree of heat stress varies with climate characteristics, as humidity plays a larger role in physiological stress in areas with high humidity, while the ambient temperature is more influential in areas with semi-arid climates [20]. Thus, different formulas for calculating THI have been developed for different climates. Temperature-humidity indices (THIs) were calculated using eight different formulas, while eight coefficients of determination (r^2^) were determined based on both THI values and the rectal temperature (RT) of 1,280 Holstein cows [23]. The r^2^ values ranged from 0.42 to 0.43 for seven of the eight THIs, while the other r^2^ was 0.39, indicating that the THIs vary depending on the formula used, and that the formula for calculating THI should be selected according to the environmental conditions [23].

At present, two formulas for calculating THI are commonly used in dairy production. One equation uses dry and wet bulb temperatures to calculate THI: THI = 0.72(Tdb+Twb)+40.6, where Tdb is the dry bulb temperature and Twb is the wet bulb temperature in °C [24]. The other equation is THI = (1.8×T+32)–(0.55–0.0055×RH9×[1.8×T–269]), where T is dry bulb temperature (°C) and RH is the relative humidity (%) [25]. According to the local climate characteristics, we can select a suitable equation for THI to ascertain potential heat stress in dairy cows, and to provide a basis for the effective prevention of heat stress on a given farm. Armstrong [26] showed that cows do not experience heat stress when the THI value is less than 72, they experience mild heat stress when 73<THI<79, they suffer moderate heat stress when 80<THI <89, and they exhibit a severe heat stress response when THI >90. Therefore, if THI can be divided into different stages according to the possible state of heat stress, this heat stress—and the degree of stress that heat and humidity may cause—can be inferred by the change in ambient temperature and humidity. Thus, heat stress in dairy cows can be subsequently reduced or alleviated via corresponding measures (e.g., such as equipping facilities with a sprinkler system and/or a fan to control environmental conditions) [6,9].

### Effects on the body temperature of dairy cows

The body temperature of cows can be measured in terms of RT, vaginal temperature (VT), rumen temperature, subcutaneous tissue temperature, and temperature measured via the ear canal [27–29]. In addition, milk temperature has also been measured to infer the body temperature of a cow [9]. In production practice, RT is often used as a sensitive index to determine whether a cow has reached thermal balance [30] and to determine the influence of the thermal environment on growth, lactation, and reproduction in dairy cows [31]. A study by Xue et al [32] showed that RT rose from 37.8°C to 38.5°C when the THI value rose from 45 to 72, representing increases in THI and RT of 60% and 1.8%, respectively. However, when THI rose from 72 to 79, RT increased from 38.5°C to 39.35°C, representing increases in THI and RT of 9.7% and 2.2%, respectively. In other words, the percent increase in RT was greater when THI was greater than 72, indicating that the correlation between RT and THI is complex. Therefore, THI less than 72 is considered to be thermoneutral zone for dairy cows [33,34]. Wen [6] showed that RT was significantly higher during heat stress than during non-heat stress periods (p<0.01), and that for each unit increase in the THI value, the RT of dairy cows increased by 0.1062°C, 0.0739°C, and 0.0847°C during early, mid-, and late lactation, respectively. In addition, the p values of correlations between THI and RT in early, mid-, and late lactation were 0.0069, 0.0292, and 0.0093, respectively, indicating that THI significantly affects the RT of dairy cows in early, mid-, and late lactation (p<0.05) [6]. Rejeb et al [35] also showed that the correlation coefficient (r) between THI and the RT of thirteen multiparous cows was as high as 0.87 when the THI value ranged from 65.62 to 83.27.

Because milk temperature is easier to monitor than RT, the relationship between milk temperature and THI has also been studied. West et al [9] showed that the milk temperature fluctuated from 39.3°C to 39.6°C (in Holsteins) and 39.1°C to 39.2°C (in Jerseys) when THI ranged between 72.1 and 83.6. Furthermore, the r value of the THI and milk temperature of Holstein and Jersey cows were 0.88 and 0.60, respectively, indicating that the milk temperature of the Holstein cows was affected more than that of the Jersey cows by THI. In addition, these results showed that milk temperature gradually increased with ambient temperature, reaching 39°C when the THI reached 72, which is the point at which the feed intake and milk production of cows declined and other heat-stress symptoms appeared, indicating that milk temperature can be effectively used to assess heat stress [9].

The relationship between VT and ambient temperature and humidity has also drawn attention, as a strong correlation between VT and RT has already been established [36,37]. The VT of 11 Holstein-Friesian cows (including six in a hot period and five in a cool period) were continuously monitored using data-recording thermal sensors for 25 days, while the ambient temperature and humidity were also continuously recorded. Nabenishi et al [29] study showed that the average VT (39.1°C) during the hot period was significantly higher than that (38.7°C) during the cool period (p<0.01). The average THI during the hot period (78.0) was also higher than that of the cool period (56.6; p<0.01). The r value between VT and maximum THI (mTHI) was 0.30 when THI ranged from 72.0 to 81.1 during the hot period, but was −0.08 when THI ranged from 48.0 to 68.8 in the cool period, indicating that VT is not substantially affected by temperature and humidity during cool periods [29]. With the development of sensing technology, the ability to continuously measure the VT of cows has been developed. As a result, the importance of VT has become increasingly prominent, where correlations between VT and RT have garnered more and more attention. A recent study on the VT and RT of 75 primiparous and 142 multiparous Holstein cows showed that, under the same THI conditions, the mean VT and RT of primiparous cows were 39.7°C and 39.4°C, respectively, while the mean VT and RT of multiparous cows were 39.5°C and 39.2°C, respectively. However, the correlations between THI and VT and RT were not explored further [38].

At present, many reports have focused on the effects of THI on the body temperature of dairy cows, where the relationships between THI and body temperature vary depending on the method used to measure body temperature [35,39]. Although RT [30], rumen temperature [40], milk temperature [9], and VT [29] can all be used to assess heat stress, VT is affected by external factors to a lesser extent than other measurements and may be more suitable for the evaluation of heat stress in dairy cows [41]. In addition to improving the heat stress assessment index [42], it is still not entirely clear if other temperature indices can be used to assess heat stress, or if the combination of body temperature and environmental indices could provide an accurate evaluation of heat stress. Therefore, further research is necessary to elucidate these possibilities.

## EFFECTS OF HEAT STRESS ON PRODUCTION AND REPRODUCTION IN DAIRY COWS

Under normal conditions, fluctuations in the body temperature of cows are very small. The normal body temperature ranges from 38.5°C to 39.5°C in calves, from 38.0°C to 39.5°C in heifers, and from 38.0°C to 39.0°C in adult cows [43]. While an ambient temperature between 0°C and 25°C has been showed to be beneficial to production performance [44,45], ambient temperatures in excess of 25°C have been shown to cause an increase in the body temperature of dairy cows, thereby increasing heat stress [45,46]. A study by Robinson et al [47] showed that heat production was 124.5 kcal/kg of BW^0.75^ when beef cattle were exposed to an ambient temperature of 20°C, but was 141.8 kcal/kg of BW^0.75^ when they were exposed to hotter conditions (35°C). This indicates that heat production increases with an increase in ambient temperature, resulting in a concomitant increase in the body temperature of the cow. However, body temperatures that exceed normal values are not ideal, and cows have been shown to decrease their feed intake and heat exchange capacity accordingly [48,49]. As a result, this leads to lower milk production and reproductive indices [24], as well as increased costs for the dairy industry in the summer months [34].

### Effects on feed intake

A large amount of metabolic heat is produced by digesting roughage, which increases the body temperature of cows. As ambient temperature increases in the summer months, and as body temperature concomitantly increases, cows decrease their feed intake to mitigate heat stress [19], thereby leading to a gradual decline in milk production and a change in milk content. It has been reported that the feed intake of dairy cows begins to decrease when the ambient temperature reaches 25°C, and sharply decreases when the ambient temperature exceeds 40°C, after which feed intake is approximately 20% to 40% lower than the normal intake [50,51]. Thus, an observed decrease in feed intake is an important index of heat stress in dairy cows, where the combination of decreased feed intake and increased ambient temperature gradually result in lower milk yield [32]. If environmental factors can be actively monitored and management practices altered accordingly to alleviate heat stress in dairy cows, milk yield and quality can be improved by boosting feed intake [4].

A study of 22 Holstein and eight Jersey cows found that THI negatively affected feed intake, where a THI>72.1 resulted in a decrease in the feed intake of Holstein and Jersey cows by 0.51 kg and 0.47 kg, respectively, for each unit of increase in THI [9]. In addition, Xue et al [32] found that the dry matter intake (DMI) increased from 18.5 to 19.8 kg/d when THI increased from 42 to 68, but decreased from 19.8 to 15.8 kg/d when the THI increased from 68 to 80. This indicates that DMI responds to THI in a biphasic manner, where DMI increases slowly with an increase in THI until a critical point, after which it decreases sharply with an increase in THI when the THI value is higher. Therefore, cows gradually enter a state of heat stress when THI exceeds 68, and DMI decreases gradually with increasing heat stress.

### Effects on milk yield

As previously mentioned, heat stress leads to a decrease in feed intake of dairy cows [19], thereby leading to a reduction in milk production [13]. Gantner et al [34] showed that the THI values in spring and summer in the three regions (east, Mediterranean, and central) of Croatia easily exceeded 72, during which the cows were prone to develop heat stress. However, in autumn and winter, when THI values were usually less than 72, the cows rarely experiencing heat stress. In addition, the milk yields were significantly different between the heat-stress and non-heat-stress periods (p<0.01), and indicating that the milk yield of dairy cows in regions with different microclimates can be significantly affected when THI reaches heat-stress levels. A study by West et al [9] that investigated the effects of heat stress on different breeds of cows, showed that the milk yield of Holstein cows (n = 22) and Jersey cows (n = 8) decreased by 0.69 kg and 0.45 kg, respectively, for every unit increase in THI, indicating that a higher degree of breed selection led to a greater impact of heat stress. Moreover, a study that investigated seasonal effects on milk yield showed that the milk yield of Holstein cows decreased by 10% to 40% in summer in comparison to the milk yield in winter [52], further highlighting the influence of heat stress on milk production.

Heat stress not only decreases milk yield, but also affects milk content and somatic cell count [53,54]. An analysis of milk produced by 1,675,686 cows in Croatia from January 2005 to April 2010 showed that the fat and protein content of the milk decreased significantly as a result of increased THI (p< 0.01) [34]. A separate analysis—based on 119,337 milk-production values from 15,012 dairy cows in Georgia, USA—showed that the fat and protein content of milk decreased by 0.012 kg and 0.009 kg, respectively, for each unit of increase in THI above 72 [13]. Yet another study regarding milk components in the Mediterranean showed that, when the average THI increased from 68 to 78 (from spring to summer), the protein and fat contents of milk decreased from 2.96% and 3.58% to 2.88% and 3.24%, respectively. In addition, feed intake and milk yield decreased by 9.6% and 21%, respectively [33]. These studies illustrate that the performance of dairy cows in summer is greatly affected by heat stress, which is indirectly reflected in changes in feed intake, milk yield, and milk components [33,55].

### Effects on follicular development and estrus

Heat stress can affect the function of tissues and organs in dairy cows and hinder the synthesis of some proteins and hormones, which can in turn lead to low fertility by affecting the synthesis of proteins and hormones associated with the reproductive organs [56]. First, heat stress has been shown to exert a direct effect on follicular development in dairy cows [57]. Wolfenson et al [58] showed that the number of dominant ovarian follicles in a heat-stressed group of dairy cows began to decline earlier than in the cooled group during the first follicular wave. The number of large follicles (diameter ≥10 mm) was significantly higher in the heat-stressed group than in the cooled group (p<0.01), resulting in 53% more large follicles in the heat-stressed group during the first follicular wave. Moreover, during the second follicular wave, the numbers of small and medium follicles were higher in the cooled group than in the heat-stressed group, and the number of medium follicles was significantly lower than that in the first follicular wave (p<0.02). In addition, the dominant ovarian follicles appeared earlier and were fewer in number than in the cooled group, indicating that heat stress can affect the development of follicles in dairy cows [58]. However, Wilson et al [59] showed that the dominant ovarian follicles in the heat-stressed group were equal in size to or smaller than those of the thermoneutral group during the second follicular wave, and that the percentage of cows having two follicular waves in the heat-stressed group and thermoneutral group was 18% and 91%, respectively. The serum concentrations of progesterone were similar for heat-stressed and thermonutral group until d 16. After d 16, serum progesterone content was significantly higher in the heat-stressed group than that in the thermoneutral group (p< 0.01) [59]. In addition, the day of functional luteolysis (defined as serum progesterone <1 ng/mL) was delayed in four out of 11 heat-stressed cows (day 29.1±2.4) in comparison to that of the thermoneutral cows (day 20.4± 2.4; p<0.05) [59]. Collectively, these studies all indicate that follicular development is adversely affected by heat stress.

In addition to affecting follicular development, heat stress also affects estrus [60]. A study on non-lactating Japanese Black cows indicated that their estrus cycle was significantly longer in summer than in winter (p<0.05), and that walking activity was also significantly lower in summer than in winter (p<0.001). However, there was no significant difference in the duration of estrous between the two seasons [41]. In addition, the estrus and conception rates decreased when THI exceeded 72 [22], indicating that heat stress increased feeding costs by reducing reproduction efficiency.

### Effects on pregnancy

Heat stress also has a serious impact on pregnancy in dairy cows [61]. A statistical analysis of pregnancies in 20,606 cows from seven farms showed that the pregnancy rates were 39.4% (THI<72.0), 38.5% (72.0 to 73.9), 36.9% (74.0 to 75.9), 32.5% (76.0 to 77.9), and 31.6% (>78.0) [15]. This indicates that the pregnancy rate of dairy cows decreases in a THI-dependent way, when THI was great than 72.0, with a decrease in pregnancy rate of 1.03% per unit increase in THI [15]. The effects of heat stress on pregnancy rate were explored in another study involving 1,199 crossbred dairy cows for 30 days before and after insemination. In this study, according to whether the ambient THI values less than 72 or not, the cows were divided into four groups: thermoneutral-thermoneutral (TN-TN), thermoneutral-heat stress (TN-HS), heat stress-thermoneutral (HS-TN), and heat stress-heat stress (HS-HS). The results showed that the pregnancy rate in the HS-HS group (20.5%) was significantly lower than those of the TN-TN (32.6%), TN-HS (30.3%), and HS-TN (31.8%) groups (p<0.05), while there were no significant differences between the latter three groups (p>0.05) [62]. These studies show that the pregnancy rate of dairy cows was decreased in response to heat stress, and that the influence of heat stress was enhanced by increased duration and intensity. However, another study showed that pregnancy rate was significantly higher after embryo transfer than that after artificial insemination (day 21, 47.6% vs 18.0%; days 45 to 60, 29.2% vs 13.5%; p<0.001) [63]. Related studies reported that heat stress negatively affects the development of bovine oviduct [64] and oocyte quality [65]. Therefore, the decline of pregnancy rate after artificial insemination may be due to the decreases in oocyte quality and oviductal function in response to heat stress.

## CONCLUSION

The body temperature and feed intake of dairy cows are affected by rising ambient temperature and humidity, which in turn affect milk production and reproduction. Although THI integrates ambient temperature and humidity information to assess heat stress, there are still many shortcomings in the research performed to date. First, the precision of THI monitoring was not sufficient. Many studies only used seasonal averages, where only a few working days were monitored. Moreover, just a few time points (e.g., morning, noon, and night) were used when monitoring working days. As a result, the results of monitoring were intermittent, did not record circadian changes in THI, and could not timely and precisely reflect the heat stress status of the cows. Second, it is not adequate for all regions and farms to determine the state of heat stress according to whether THI exceeds 68 or 72. Instead, specific problems should be analyzed more concretely. Third, heat stress is the physiological reaction of a given cow to its thermal environment, and is not only affected by THI, but also varies between breeds, individuals, farms, and management practices. Thus, inferring heat stress using only THI is not sufficiently analytical. The magnitude of heat stress should be more accurately reflected using a comprehensive assessment of environmental index and physiological parameter. With the rapid development of mechanical and electrical sensing technology, automatic monitoring equipment for the body temperatures and activities of cows has been gradually improved [29,66]. Moving forward, these techniques will be important in the scientific evaluation of heat stress in cows by combining THI, body temperature, and other indices (e.g., activity) to make the management of dairy cows more refined, and even individualized, to effectively forecast heat stress.

## References

[1] Yang PG (2014). Effects of heat stress on meat quality and muscle metabolites of finishing pigs.

[2] Yang YL, Ye BK, Liu HY (2010). Occurrence, danger, prevention and treatment of heat stress in dairy cattle. China Cattle Sci.

[3] Mader TL, Davis MS (2004). Effect of management strategies on reducing heat stress of feedlot cattle: feed and water intake. J Anim Sci.

[4] Fournel S, Ouellet V, Charbonneau É (2017). Practices for alleviating heat stress of dairy cows in humid continental climates: a literature review. Animals.

[5] Renaudeau D, Collin A, Yahav S, De Basilio V, Gourdine JL, Collier RJ (2012). Adaptation to hot climate and strategies to alleviate heat stress in livestock production. Animal.

[6] Wen YL (2011). Effects of heat stress on performance and physiological functions in dairy cows.

[7] Novak P, Vokralova J, Broucek J (2009). Effects of the stage and number of lactation on milk yield of dairy cows kept in open barn during high temperatures in summer months. Arch Anim Breed.

[8] Mohammed AN, Aziz RLA, Zeinhom MMA (2015). Exploitation of multiple approaches to adapt and mitigate the negative effects of heat stress on milk production and fertility of Fresian cows under field conditions. J Vet Med Sci.

[9] West JW, Mullinix BG, Bernard JK (2003). Effects of hot, humid weather on milk temperature, dry matter intake, and milk yield of lactating dairy cows. J Dairy Sci.

[10] Hicks L, Hicks W, Bucklin R (2001). Comparison of methods of measuring deep body temperature of dairy cows.

[11] Brown-Brandl TM, Eigenberg RA, Nienaber JA, Hahn GL (2005). Dynamic response indicators of heat stress in shaded and non-shaded feedlot cattle, part 1: analyses of indicators. Biosyst Eng.

[12] Collier RJ, Dahl GE, VanBaale MJ (2006). Major advances associated with environmental effects on dairy cattle. J Dairy Sci.

[13] Ravagnolo O, Misztal I, Hoogenboom G (2000). Genetic component of heat stress in dairy cattle, development of heat index function. J Dairy Sci.

[14] Lambertz C, Sanker C, Gauly M (2014). Climatic effects on milk production traits and somatic cell score in lactating Holstein-Friesian cows in different housing systems. J Dairy Sci.

[15] Lozano Domínguez RR, Vásquez Peláez CG, Padilla EG (2005). Effect of heat stress and its interaction with other management and productive variables on pregnancy rate in dairy cows in Aguascalientes, Mexico. Vet Max.

[16] St-Pierre NR, Cobanov B, Schnitkey G (2003). Economic losses from heat stress by US livestock industries. J Dairy Sci.

[17] Li XJ, Wang ZL, Chen XL (2016). Study progress on the rule of body temperature and its application in reproduction of dairy cattle. Acta Vet Zootech Sin.

[18] Kou HX, Zhao FP, Ren K (2016). The progress on detection method and the regularities of body temperature and activities in dairy cows. Acta Vet Zootech Sin.

[19] Ammer S, Lambertz C, von Soosten D (2018). Impact of diet composition and temperature–humidity index on water and dry matter intake of high-yielding dairy cows. J Anim Physiol Anim Nutr (Berl).

[20] Bohmanova J, Misztal I, Cole JB (2007). Temperature-humidity indices as indicators of milk production losses due to heat stress. J Dairy Sci.

[21] Prunier A, Messias De Bragança M, Le Dividich J (1997). Influence of high ambient temperature on performance of reproductive sows. Livest Prod Sci.

[22] Cartmill JA, El-Zarkouny SZ, Hensley BA, Rozell TG, Smith JF, Stevenson JS (2001). An alternative AI breeding protocol for dairy cows exposed to elevated ambient temperatures before or after calving or both. J Dairy Sci.

[23] Dikmen S, Hansen PJ (2009). Is the temperature-humidity index the best indicator of heat stress in lactating dairy cows in a subtropical environment?. J Dairy Sci.

[24] Kadzere CT, Murphy MR, Silanikove N, Maltz E (2002). Heat stress in lactating dairy cows: a review. Livest Prod Sci.

[25] Legrand A, Schütz KE, Tucker CB (2011). Using water to cool cattle: behavioral and physiological changes associated with voluntary use of cow showers. J Dairy Sci.

[26] Armstrong DV (1994). Heat stress interaction with shade and cooling. J Dairy Sci.

[27] Ipema AH, Goense D, Hogewerf PH, Houwers HWJ, van Roest H (2008). Pilot study to monitor body temperature of dairy cows with a rumen bolus. Comput Electron Agric.

[28] Lee Y, Bok JD, Lee HJ (2016). Body temperature monitoring using subcutaneously implanted thermo-loggers from holstein steers. Asian-Australas J Anim Sci.

[29] Nabenishi H, Ohta H, Nishimoto T, Morita T, Ashizawa K, Tsuzuki Y (2011). Effect of the Temperature-humidity index on body temperature and conception rate of lactating dairy cows in southwestern Japan. J Reprod Dev.

[30] Younes, Moez, Ayadi (2011). Hormonal (thyroxin, cortisol) and immunological (leucocytes) responses to cistern size and heat stress in Tunisia. J Life Sci.

[31] West JW (1999). Nutritional strategies for managing the heat-stressed dairy cow. J Anim Sci.

[32] Xue B, Wang ZS, Li SL, Wang LZ, Wang ZX (2010). Temperature-humidity index on performance of cows. China Anim Husb Vet Med.

[33] Bouraoui R, Lahmar M, Majdoub A, Djemali M, Belyea R (2002). The relationship of temperature-humidity index with milk production of dairy cows in a Mediterranean climate. Anim Res.

[34] Gantner V, Mijić P, Kuterovac K, Solić D, Gantner R (2011). Temperature-humidity index values and their significance on the daily production of dairy cattle. Mljekarstvo.

[35] Rejeb M, Sadraoui R, Najar T, Ben MM, Rejeb M (2016). A complex interrelationship between rectal temperature and dairy cows’ performance under heat stress conditions. Open J Anim Sci.

[36] Suthar V, Burfeind O, Maeder B, Heuwieser W (2013). Agreement between rectal and vaginal temperature measured with temperature loggers in dairy cows. J Dairy Res.

[37] Vickers LA, Burfeind O, von Keyserlingk MAG, Veira DM, Weary DM, Heuwieser W (2010). Technical note: Comparison of rectal and vaginal temperatures in lactating dairy cows. J Dairy Sci.

[38] Burfeind O, Suthar VS, Voigtsberger R, Bonk S, Heuwieser W (2014). Body temperature in early postpartum dairy cows. Theriogenology.

[39] Gonzalez-Rivas PA, Sullivan M, Cottrell JJ, Leury1 BJ, Gaughan JB, Dunshea FR (2016). A rumen bolus is a useful tool to monitor core body temperature in lactating dairy cows in a sub-tropical summer. J Anim Sci.

[40] Lees AM, Lees JC, Lisle AT, Sullivan ML, Gaughan JB (2018). Effect of heat stress on rumen temperature of three breeds of cattle. Int J Biometeorol.

[41] Sakatani M, Balboula AZ, Yamanaka K, Takahashi M (2012). Effect of summer heat environment on body temperature, estrous cycles and blood antioxidant levels in Japanese Black cow. Anim Sci J.

[42] Mader TL, Johnson LJ, Gaughan JB (2010). A comprehensive index for assessing environmental stress in animals. J Anim Sci.

[43] Ma YL, Du WK (2010). Relationship between body temperature and disease of cattle. Shangdong J Anim Sci Vet Med.

[44] McDowell RE (1972). Improvement of livestock production in warm climates.

[45] Correa-Calderon A, Armstrong D, Ray D, DeNise S, Enns M, Howison C (2004). Thermoregulatory responses of Holstein and Brown Swiss Heat-Stressed dairy cows to two different cooling systems. Int J Biometeorol.

[46] Bitman J, Lefcourt A, Wood DL, Stroud B (1984). Circadian and ultradian temperature rhythms of lactating dairy cows. J Dairy Sci.

[47] Robinson JB, Ames DR, Milliken GA (1986). Heat production of cattle acclimated to cold, thermoneutrality and heat when exposed to thermoneutrality and heat stress. J Anim Sci.

[48] Könyves T, Zlatković N, Memiši N (2017). Relationship of temperature-humidity index with milk production and feed intake of holstein-frisian cows in different year seasons. Thai J Vet Med.

[49] Gorniak T, Meyer U, Südekum K-H, Dänicke S (2014). Impact of mild heat stress on dry matter intake, milk yield and milk composition in mid-lactation Holstein dairy cows in a temperate climate. Arch Anim Nutr.

[50] Hahn GL (1999). Dynamic responses of cattle to thermal heat loads dynamic responses of cattle to thermal heat loads. J Anim Sci.

[51] Zang CJ (2008). Effect of dietary cation-anion balance on production performance and blood biochemistry indicators of diary cows in the condition of heat stress.

[52] Du Preez JH, Giesecke WH, Eisenberg BE (1990). Heat stress in dairy cattle and other livestock under southern African conditions. III. Monthly temperature-humidity index mean values and their significance in the performance of dairy cattle. Onderstepoort J Vet Res.

[53] Bertocchi L, Vitali A, Lacetera N, Nardone A, Varisco G, Bernabucci U (2014). Seasonal variations in the composition of Holstein cow’s milk and temperature-humidity index relationship. Animal.

[54] Nasr MAF, El-Tarabany MS (2017). Impact of three THI levels on somatic cell count, milk yield and composition of multiparous Holstein cows in a subtropical region. J Therm Biol.

[55] Herbut P, Angrecka S (2012). Forming of temperature-humidity index (THI) and milk production of cows in the free-stall barn during the period of summer heat. Anim Sci Pap Rep.

[56] Putney DJ, Malayer JR, Gross TS, Thatcher WW, Hansen PJ, Drost M (1988). Heat stress-induced alterations in the synthesis and secretion of proteins and prostaglandins by cultured bovine conceptuses and uterine endometrium. Biol Reprod.

[57] Badinga L, Thatcher WW, Diaz T, Drost M, Wolfenson D (1993). Effect of environmental heat stress on follicular development and steroidogenesis in lactating Holstein cows. Theriogenology.

[58] Wolfenson D, Thatcher WW, Badinga L (1995). Effect of heat stress on follicular development during the estrous cycle in lactating dairy cattle. Biol Reprod.

[59] Wilson SJ, Marion RS, Spain JN, Spiers DE, Keisler DH, Lucy MC (1998). Effects of controlled heat stress on ovarian function of dairy cattle. 1. Lactating cows. J Dairy Sci.

[60] Sakatani M, Takahashi M, Takenouchi N (2016). The efficiency of vaginal temperature measurement for detection of estrus in Japanese Black cows. J Reprod Dev.

[61] Ingraham RH, Gillette DD, Wagner WD (1974). Relationship of temperature and humidity to conception rate of holstein cows in subtropical climate. J Dairy Sci.

[62] Khan FA, Prasad S, Gupta HP (2013). Effect of heat stress on pregnancy rates of crossbred dairy cattle in Terai region of Uttarakhand, India. Asian Pacific J Reprod.

[63] Putney DJ, Drost M, Thatcher WW (1989). Influence of summer heat stress on pregnancy rates of lactating dairy cattle following embryo transfer or artificial insemination. Theriogenology.

[64] Kobayashi Y, Wakamiya K, Kohka M, Yamamoto Y, Okuda K (2013). Summer heat stress affects prostaglandin synthesis in the bovine oviduct. Reproduction.

[65] Al-Katanani YM, Paula-Lopes FF, Hansen PJ (2002). Effect of season and exposure to heat stress on oocyte competence in holstein cows. J Dairy Sci.

[66] Kou HX, Zhao YQ, Ren K, Chen XL, Lu YQ, Wang D (2017). Automated measurement of cattle surface temperature and its correlation with rectal temperature. PLoS One.

